# Feasibility of scenario-based simulation training versus traditional workshops in continuing medical education: a randomized controlled trial

**DOI:** 10.3402/meo.v18i0.21312

**Published:** 2013-07-18

**Authors:** Brendan Kerr, Trisha Lee-Ann Hawkins, Robert Herman, Sue Barnes, Stephanie Kaufmann, Kristin Fraser, Irene W. Y. Ma

**Affiliations:** 1 Department of Medicine, University of Calgary, Calgary, AB, Canada; 2 e-SIM, Provincial Simulation Program, Alberta Health Services, Calgary, AB, Canada; 3 W21C, University of Calgary, Calgary, AB, Canada

**Keywords:** patient simulation, continuing medical education, pregnancy-induced hypertension

## Abstract

**Introduction:**

Although simulation-based training is increasingly used for medical education, its benefits in continuing medical education (CME) are less established. This study seeks to evaluate the feasibility of incorporating simulation-based training into a CME conference and compare its effectiveness with the traditional workshop in improving knowledge and self-reported confidence.

**Methods:**

Participants (*N*=27) were group randomized to either a simulation-based workshop or a traditional case-based workshop.

**Results:**

Post-training, knowledge assessment score neither did increase significantly in the traditional group (*d*=0.13; *p*=0.76) nor did significantly decrease in the simulation group (*d*= − 0.44; *p*=0.19). Self-reported comfort in patient assessment parameters increased in both groups (*p*<0.05 in all). However, only the simulation group reported an increase in comfort in patient management (*d*=1.1, *p*=0.051 for the traditional group and *d*=1.3; *p*= 0.0003 for the simulation group). At 1 month, comfort measures in the traditional group increased consistently over time while these measures in the simulation group increased post-workshop but decreased by 1 month, suggesting that some of the effects of training with simulation may be short lived.

**Discussion:**

The use of simulation-based training was not associated with benefits in knowledge acquisition, knowledge retention, or comfort in patient assessment. It was associated with superior outcomes in comfort in patient management, but this benefit may be short-lived. Further studies are required to better define the conditions under which simulation-based training is beneficial.

## Introduction

In order for professional self-regulation to continue, physicians’ participation in lifelong learning is the foundation that underlies society's trust in the profession (1). Continuing medical education (CME) in the form of participation in certified educational activities is a widely recognized method to ensure that physicians maintain their medical knowledge and skills (2). However, multiple studies have questioned the effectiveness of CME activities in modifying physician behaviors and performance, and those in the format of didactic lectures, in particular, were found to be largely ineffective (3–5). Nonetheless, CME activities are not entirely without merit (6–8). What has consistently emerged in the literature is the notion that interactive or mixed CME activities can be effective in modifying physician practices (9, 10).

Simulation is an interactive educational tool that is increasingly used in medical education (11) and there is mounting evidence to support its role in improving knowledge, behaviors, and product skill outcomes (12–14). Yet despite this evidence, simulation is seldom discussed within the CME setting (15). The adoption of new technology into the CME arena may be limited, in part, by cultural barriers and forces of inertia (15). Given a choice, physicians tend to prefer traditional passive modalities of education, such as the didactic lecture (16–18). In addition, the usual limitations of and barriers to implementation of simulation-based education apply equally to the non-CME setting as to the CME setting, in that this form of learning can be expensive and cumbersome to deliver. In view of the potential reluctance to adopt new technology into CME activities, this pilot study seeks to evaluate the feasibility of incorporating simulation-based training into an existing CME conference and discuss the role of simulation-based CME education with respect to the currently available evidence, feasibility, and future research directions.

## Methods

### Participants

All consenting attendees of the 2011 Rocky Mountain/American College of Physicians Internal Medicine Meeting enrolled in the ‘Assessment of Shortness of Breath in Pregnancy’ workshop were included in the study. Participants included academic- and community-based general internists. A minority of participants, approximately 30%, were residents.

Participants were group randomized to either a simulation-based workshop or a traditional case-based interactive workshop. Both groups were exposed to the same obstetric-internal medicine case (shortness of breath in pregnancy, secondary to pulmonary edema from severe pre-eclampsia) by the same workshop leaders. The duration of the workshop for both groups was 1 h. This study was approved by the University of Calgary Conjoint Health Research Ethics Board.

### Simulation-based workshop

Participants in the simulation group were provided with a 5-min orientation and an introduction to the features of the computer-based pregnant mannequin (Noelle^®^ S575, Gaumard^®^, Miami, FL, USA). The patient's clinical status was controlled remotely using a wireless tablet, with patient's vital signs displayed on a touchscreen monitor at the bedside. Participants were encouraged to work as a group. One nurse confederate assisted in running the scenario. The duration of the interactive case was 30 min, followed by a 15-min debriefing session and a 10-min didactic session covering the key teaching points of the workshop.

### Traditional case-based workshop

Participants in the traditional group participated in a case-based interactive workshop. The same obstetric-internal medicine case as the simulation-based workshop was discussed in a 55-min session by the same workshop leaders, covering the same key teaching points as the simulation-based workshop.

### Outcome assessment

Participants from both groups completed a baseline survey and knowledge assessment pre- and immediately post-workshop. Knowledge retention was assessed at 1-month with an internet-based knowledge assessment tool.

Knowledge assessment pre- and post-workshop was done using two parallel forms, each containing 10 multiple-choice questions (MCQ). Retention knowledge assessment used the same form as the post-workshop MCQs. MCQs were constructed by an expert panel based on a table of specifications, covering items on pathophysiology, epidemiology, diagnosis, and management, and assessed for knowledge comprehension and knowledge application. The expert panel consisted of one specialist in obstetric-internal medicine and two specialists in general internal medicine. The MCQs were piloted on and revised based on input on appropriateness of content, wording clarity, and difficulty level from internal medicine residents (*N*=3) and faculty with maternal–fetal medicine expertise (*N*=1).

### Statistical analysis

Comparisons between groups were performed using standard parametric and non-parametric tests: Student's *t*-tests, paired *t*-tests, Wilcoxon's rank sum tests, Fisher's exact tests, and Chi-square tests. Measures of effect size are reported using Cohen's *d* (19).

Repeated-measures mixed-factor analyses of variance (ANOVA) were conducted to assess whether there were group and score differences, after examination of normality and sphericity using Mauchly's test of sphericity.

All reported *p*-values are two-sided. For all *post-hoc* multiple comparisons between groups, the nominal α level was adjusted by Bonferroni correction. All analyses were performed using SAS version 9.3 (SAS Institute Inc, Cary, NC, USA) and PASW Statistics Software, version 18.0 for Windows (PASW, IBM Corporation, Somers, NY, USA).

## Results

**Fig. 1:**
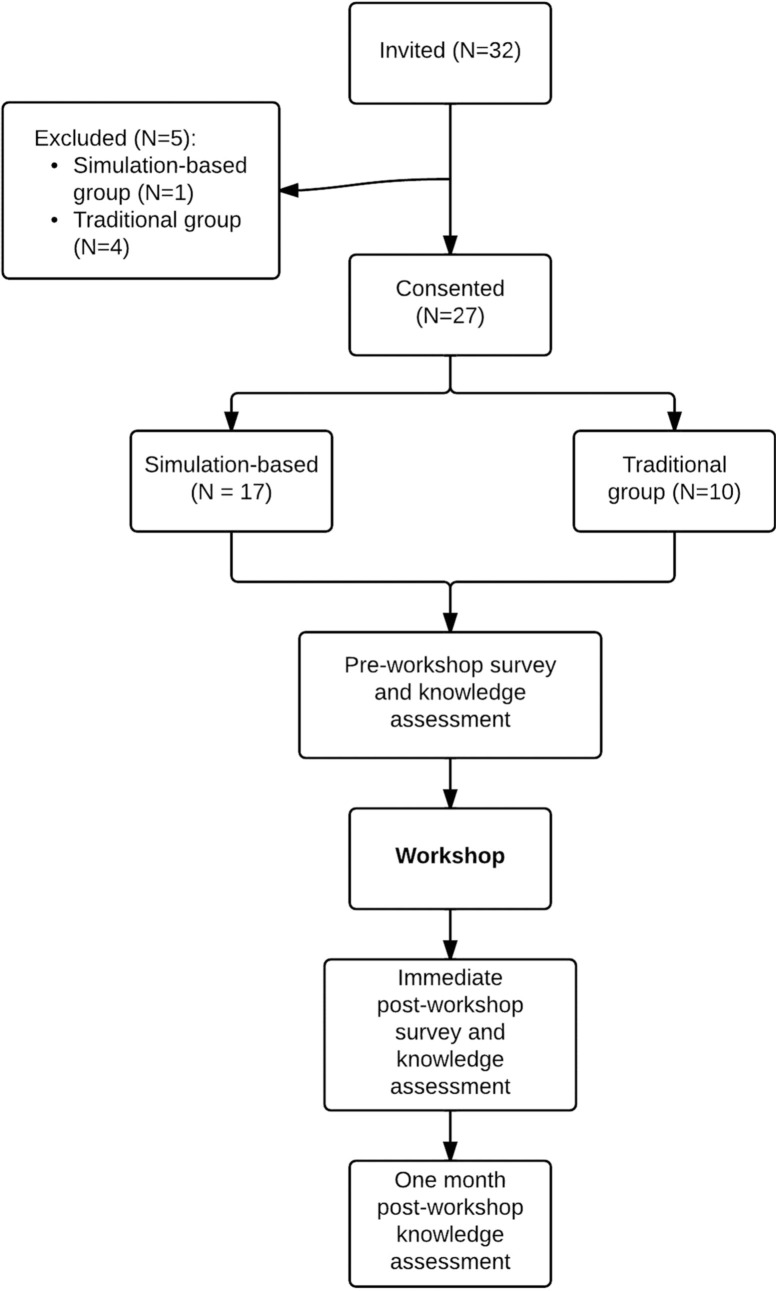
Overview of study design.

Of the 32 participants invited, 27 consented to the study. In total, three 1-h workshops were conducted. Of the 27 participants, a group of 10 was randomized to the traditional group, while a group of 9 and a group of 8 were randomized to the simulation group (Fig. 1).

As demonstrated in Table 1, there were no significant baseline differences between the two groups with the exception of one variable. Participants in the simulation group at baseline felt that simulation was more valuable for medical teaching (mean 4.7±0.5) compared to the traditional group (mean 4.2±0.4; *p*<0.01).

**Table 1:** Baseline characteristics of workshop participants

	Traditional (*N*=10)	Simulation (*N*=17)	*p*
Males – number (%)	3 (30)	7 (41)	0.69
Duration of practice			
1–5 years – number (%)	2 (20)	0	0.33
6–10 years – number (%)	0	0	
11–15 years – number (%)	1 (10)	1 (6)	
16–20 years – number (%)	0	0	
21 years or more – number (%)	1 (10)	2 (12)	
Not yet in practice – number (%)	6 (60)	14 (82)	0.36
University-based – number (%)	5 (50)	15 (75)	0.14
Received additional training in Obstetric Internal Medicine – number (%)	1 (10)	4 (24)	0.62
Previously learned skills using high-fidelity simulation – number (%)	6 (60)	11 (65)	1.00
I am comfortable participating in the care of medically complicated pregnant patients*	2.2±0.8	2.5±0.9	0.45
I am comfortable evaluating a pregnant patient with shortness of breath*	2.9±1.0	2.8±0.8	0.71
I am comfortable managing shortness of breath in the pregnant patient*	2.7±0.9	2.8±0.8	0.85
I am comfortable being taught with high-fidelity simulation*	4.2±0.4	3.9±0.7	0.28
I think simulation in general is valuable for the purposes of medical teaching*	4.2±0.4	4.7±0.5	0.01

* 1 = strongly disagree; 5 = strongly agree.

### Knowledge assessment scores

Baseline MCQ scores were not different between the two groups (*p*=0.22; Table 2). Post-workshop MCQ scores were also not significantly different between the two groups (*p*=0.90; Table 2). The change in scores pre-and post-workshop was not significant for either group (*p*>0.05).

**Table 2:** Differences in measures for traditional group pre- and post-workshop and simulation group pre- and post-workshop

	Traditional Group Pre-Workshop (*N*=10) (mean±SD)	Traditional Group Post-Workshop (N = 10) (mean±SD)	Cohen's *d*; *p*-value	Simulation-based Pre-Workshop (*N*=17)	Simulation-based Post-Workshop (*N*=17) (mean±SD)	Cohen's *d*; *p*-value
Baseline knowledge assessment scores	50.0%±16.3%	52.0%±15.5%	*d*=0.13; *p*=0.76	58.8%±18.3%	51.2%±16.2%	*d*= −0.44; *p*=0.19
Self-reported comfort in *participating in the care* of medically complicated pregnant patients	2.2±0.8	3.3±0.95	*d*=1.26; *p*=0.003	2.5±0.9	3.3±0.85	*d*=0.91; *p*=0.004
Self-reported comfort in *evaluating* a pregnant patient with shortness of breath	2.9±1.0	3.8±0.63	*d*=1.1; *p*=0.02	2.8±0.8	3.9±0.33	*d*=1.8; *p*<0.001
Self-reported comfort in *managing* shortness of breath in a pregnant patient	2.7±0.9	3.3±0.95	*d*=0.63; *p*=0.051	2.8±0.8	3.6±0.61	*d*=1.3; *p*<0.001

### Self-reported comfort

Self-reported comfort in *participating in the care of medically complicated pregnant patients* increased for both groups (*d*=1.26; *p*=0.003 for the traditional group and *d*=0.91; *p*=0.004 for the simulation group; Table 2). Self-reported comfort in *evaluating a pregnant patient with shortness of breath* also significantly increased in both groups (*d*=1.1; *p*= 0.02 for the traditional group and *d*=1.8; *p*<0.001 for the simulation group; Table 2). However, only the simulation group reported a significant increase in self-reported comfort in *managing shortness of breath in a pregnant patient* (*d*=0.63; *p*=0.051 for the traditional group and *d*=1.3; *p*=0.0003 for the simulation group, Table 2).

### Retention knowledge assessment scores and self-reported comfort

Data were only available for eight participants (29.6%): four participants from the traditional group and four from the simulation group.

**Fig. 2:**
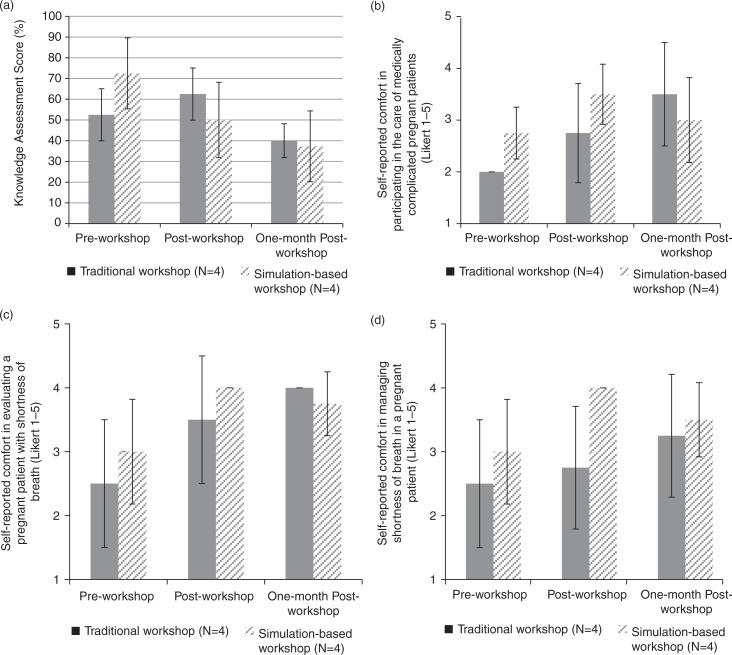
One-month retention scores of participants (*N*=8) in the traditional case-based interactive workshop and simulation-based workshop. (a) Knowledge assessment scores; (b) self-reported comfort in participating in the care of the medically complicated pregnant patient; (c) self-reported comfort in evaluating a pregnant patient with shortness of breath; and (d) self-reported comfort in managing shortness of breath in a pregnant patient.

MCQ scores increased post-workshop for the traditional group but declined at 1-month (52.5±12.6%, 62.5±12.6%, and 40.0±8.2%, respectively, Fig. 2a). MCQ score pre-workshop, post-workshop, and at 1-month decreased steadily for the simulation group (72.5±17.1%, 50.0±18.2, and 37.5±17.1%, respectively).

Self-reported comfort measures in participating in the care of medically complicated pregnant patients, evaluating a pregnant patient with shortness of breath, and managing shortness of breath in a pregnant patient all rose steadily between pre-workshop, post-workshop, and at 1-month for the traditional group (Fig. 2b–d, respectively). While these measures increased post-workshop, they decreased at 1-month for the simulation group (Fig. 2b–d, respectively).

For all retention scores, results from mixed ANOVA indicated a significant main effect of scores but no effect of group. No significant interaction between scores and group was found. *Post-hoc* analyses indicated that there were no differences in the scores between or within group (*p*>0.05 in all instances).

### Satisfaction

Although satisfaction with the workshop was higher for all items for the simulation group (Table 3), none of these differences were significant.

**Table 3:** Workshop satisfaction for both traditional and simulation groups

	Traditional (*N*=10)	Simulation (*N*=17)	*p*
Met stated objectives*	4.0±1.2	4.2±0.8	0.54
Enhanced my knowledge*	4.0±1.2	4.4±0.9	0.37
Satisfied my expectations*	4.0±1.2	4.4±0.9	0.39
Conveyed information that applied to my practice*	4.0±1.2	4.1±0.8	0.88
Allocated at least 25% of the time for interaction*	3.5±1.6	4.2±0.7	0.19
Was free from commercial bias*	4.0±1.2	4.6±0.5	0.12
Will help me practice more safely*	4.0±1.2	4.2±0.9	0.56
Will change my clinical practice*	3.8±1.1	3.9±1.0	0.73
Exposed me to new clinical situations*	3.7±1.3	4.2±1.0	0.27

* 1 = strongly disagree; 5 = strongly agree.

## Discussion

In this pilot randomized controlled study comparing the use of a traditional case-based workshop with a simulation-based workshop in a CME setting, our results suggest that although implementing a simulation-based workshop was feasible and well-received, we did not identify significant differences in knowledge acquisition, retention, self-reported comfort in medical care assessment parameters or satisfaction. The benefits of this simulation-based workshop appeared restricted only to improving comfort in management only, demonstrating a large effect size pre-and post-training (*d* =1.1). However, even this benefit of our simulation-based workshop appeared short-lived.

Our study is limited by a small sample size and it involved only one CME conference. These issues preclude definitive conclusions and generalizability about the effects of simulation-based workshops on learning outcomes in the CME setting. The pattern of results that emerged from our study should therefore only be considered as hypothesis generating. Nonetheless, these patterns of results deserve some discussion. In particular, three points regarding the use of simulation-based training in CME merit further discussion. First, what is the currently available evidence supporting the use of simulation-based CME training and how do these results fit in within the context of available evidence? Second, what is the feasibility of using simulation in a CME setting? Third, what future studies are needed to enable educators to optimally implement simulation training in the CME setting?

### Evidence

Simulation-based health education strategies range from part-task trainers and virtual reality systems to integrated scenarios with high-fidelity manikins (20). In a recent systematic review, compared to other instructional methods, simulation-based education was found to be effective (21). However, only 6% of the studies found in the review involved physicians in practice. The majority of the studies were conducted in a non-CME setting.

Because instructional strategies that are beneficial for novice learners may not always be beneficial for expert learners (expertise reversal effect) (22–24), it is important that the effectiveness for the use of simulation in the CME setting be established empirically rather than inferred from non-CME studies.

Theoretically, simulation-based education should be well suited for CME training. Experiential learning techniques, after all, are recommended based on the adult learning theory (25). Furthermore, interactivity has consistently been shown to be effective for learning in CME (26), while passive learning approaches are generally shown to be ineffective in changing behaviors (27). As simulation-based teaching techniques are generally interactive in nature, superior learning outcomes should be expected. However, emotions and cognitive load associated from participation in novel simulation-based exercises may negatively impact on learning outcomes (28).

Despite the theoretical advantages of the use of simulation, few studies have directly evaluated the use of simulation in CME (15, 29, 30), outside technical skill training (31–35). Our study indicates that simulation-based education can be feasible, and may result in similar learning outcomes. However, of particular concern is our study's finding that simulation-based training may be potentially associated with a decrement in knowledge. Although this decrement is not significant, our study sample size is small. Therefore, larger studies are needed to preclude a detrimental effect of simulation-based training on knowledge outcomes. Despite the notion that knowledge is considered the lowest level of competence in Miller's model of clinical competence (36), it is also noteworthy that knowledge is the basic foundation of competence and expertise (37). Therefore, any significant decrement in knowledge should merit concern. Additional evidence is needed to clarify the effect of simulation-based education on knowledge acquisition and knowledge application.

### Feasibility

Multiple studies have identified that practicing physicians tend to prefer passive modalities of education (16–18, 38). Amongst interactive modalities of education, simulation in particular poses additional barriers for implementation in the CME setting, such as the concern that participants may feel intimated and embarrassed (39, 40), issues regarding simulator fidelity (29, 39), and costs (37).

In a survey of physicians, only 23% felt that simulation or virtual patients as being helpful for CME (16). Anesthesiologists, however, are more receptive to the use of simulation, with up to 82% expressing an interest in simulation-based CME (40), and simulation courses in crisis resource management for anesthesia faculty have been well-received (41). Feasibility in one medical specialty, however, may not translate into feasibility in another, since medical and surgical specialists may prefer to learn in very different ways (26, 42, 43). Each specialty may have unique barriers to implementing simulation-based CME. Therefore, additional studies on feasibility in other disciplines are needed.

### Future research

Our study has identified several areas for future investigations. First, the effect of simulation-based CME activities on knowledge acquisition and application needs to be clarified. Simulation may not be the most efficient or effective modality for knowledge acquisition, since multiple non-simulation-based CME activities have been shown to effect knowledge gain (44). Second, future research should focus on the effect of simulation-based CME activities on clinically important outcomes. For example, research will benefit from the use of Moore's Outcome-based CME valuation Model, which proposes measuring outcomes on six levels: participation, satisfaction, learning, performance, patient health, and population health (45). While effects on patient health and population are the ultimate purposes of CME, research efforts should at a minimum target learning and performance outcomes (37). Third, feasibility of implementation of simulation-based CME education should be further evaluated across different disciplines.

## Conclusion

Our study demonstrated that the use of simulation-based CME education was feasible and well-received, but it failed to identify a benefit in knowledge acquisition and retention. Future studies should clarify the effect of simulation on knowledge acquisition and application as well as feasibility across different disciplines.
